# Epigenetic mapping and functional analysis in a breast cancer metastasis model using whole-genome promoter tiling microarrays

**DOI:** 10.1186/bcr2121

**Published:** 2008-07-18

**Authors:** David I Rodenhiser, Joseph Andrews, Wendy Kennette, Bekim Sadikovic, Ariel Mendlowitz, Alan B Tuck, Ann F Chambers

**Affiliations:** 1 London Regional Cancer Program, Victoria Research Laboratories, London Health Sciences Centre, 790 Commissioners Road East, London, Ontario, N6A 4L6, Canada; 2 Department of Biochemistry, The Schulich School of Medicine and Dentistry, University of Western Ontario, London, Ontario, N6A 3K7, Canada; 3 Department of Oncology, The Schulich School of Medicine and Dentistry, University of Western Ontario, London, Ontario, N6A 3K7, Canada; 4 Department of Paediatrics, The Schulich School of Medicine and Dentistry, University of Western Ontario, London, Ontario, N6A 3K7, Canada; 5 EpiGenWestern Research Group at the Children's Health Research Institute, Victoria Research Laboratories, 800 Commissioners Road East, London, Ontario, N6C 2V5, Canada; 6 Department of Pathology, The Schulich School of Medicine and Dentistry, University of Western Ontario, London, Ontario, N6A 3K7, Canada

## Abstract

**Introduction:**

Breast cancer metastasis is a complex, multi-step biological process. Genetic mutations along with epigenetic alterations in the form of DNA methylation patterns and histone modifications contribute to metastasis-related gene expression changes and genomic instability. So far, these epigenetic contributions to breast cancer metastasis have not been well characterized, and there is only a limited understanding of the functional mechanisms affected by such epigenetic alterations. Furthermore, no genome-wide assessments have been undertaken to identify altered DNA methylation patterns in the context of metastasis and their effects on specific functional pathways or gene networks.

**Methods:**

We have used a human gene promoter tiling microarray platform to analyze a cell line model of metastasis to lymph nodes composed of a poorly metastatic MDA-MB-468GFP human breast adenocarcinoma cell line and its highly metastatic variant (468LN). Gene networks and pathways associated with metastasis were identified, and target genes associated with epithelial–mesenchymal transition were validated with respect to DNA methylation effects on gene expression.

**Results:**

We integrated data from the tiling microarrays with targets identified by Ingenuity Pathways Analysis software and observed epigenetic variations in genes implicated in epithelial–mesenchymal transition and with tumor cell migration. We identified widespread genomic hypermethylation and hypomethylation events in these cells and we confirmed functional associations between methylation status and expression of the CDH1, CST6, EGFR, SNAI2 and ZEB2 genes by quantitative real-time PCR. Our data also suggest that the complex genomic reorganization present in cancer cells may be superimposed over promoter-specific methylation events that are responsible for gene-specific expression changes.

**Conclusion:**

This is the first whole-genome approach to identify genome-wide and gene-specific epigenetic alterations, and the functional consequences of these changes, in the context of breast cancer metastasis to lymph nodes. This approach allows the development of epigenetic signatures of metastasis to be used concurrently with genomic signatures to improve mapping of the evolving molecular landscape of metastasis and to permit translational approaches to target epigenetically regulated molecular pathways related to metastatic progression.

## Introduction

Metastasis is a complex, multi-step biological process characterized by distinct, interrelated steps that vary in their timing and efficiency [1,2]. These steps include the invasion of primary tumor cells into the surrounding tissue, intravasation into and through the local blood or lymphatic circulation, extravasation from the circulation and arrest of the tumor cell at a secondary site, and finally the colonization and growth of metastatic cells at that distant location [2-4]. Complex genetic and epigenetic alterations govern the efficiency of each of these steps. However, the molecular characteristics of metastasis in general, and breast cancer metastasis in particular, are primarily understood in the context of genetic changes identified with the use of gene-specific, tissue-specific and whole-genome approaches. For example, individual metastasis suppressor genes have been identified that, when lost or mutated, are permissive to the metastatic or invasive phenotype [5,6]. In addition, various microarray studies have generated genetic signatures of metastasis related to risk [7,8], clinical outcome [9] and distant recurrence [10], and have identified candidates for targeted therapy [11-13].

In contrast, epigenetic alterations in metastasis are less well characterized than these genetic changes [9,14]. Such epigenetic alterations primarily involve DNA hypermethylation events within the promoter regions of individual candidate genes. This reversible addition of methyl groups at cytosines within CpG dinucleotides can promote the recruitment of protein complexes that repress transcription and also prevent the binding of transcription factors to their binding motifs [15,16]. Additionally, the hypomethylation of repetitive elements can occur and leads to genomic instability [17,18], and alterations in histone protein modifications can have profound consequences that contribute to cancer and define an epigenetic signature of tumorigenesis [16,19,20].

Studies addressing epigenetic contributions to breast cancer metastasis have primarily focused on mapping increased DNA methylation within the promoter regions of individual candidate genes or small sets of cancer-related genes [21]. For example, hypermethylation of the E-cadherin (CDH1) promoter, and the resultant decrease in its expression, are associated with infiltrating breast cancers [22]. A limited hypermethylation profile associated with sentinel lymph node metastasis has been described that also involves significant hypermethylation in CDH1, with measureable methylation also evident in RASSF1A, RAR-β 2, APC, TWIST and GSTP1 [23]. In some cases, promoter hypermethylation has been correlated with specific tumor characteristics, such as GSTP1 methylation with increased tumor size, the occurrence of CDH1 methylation in estrogen receptor (ESR1)-negative tumors and the frequent appearance of RAR-β 2 hypermethylation in HER2-positive tumors [23]. Similar multi-gene correlations have linked metastases of breast cancer cells to sentinel lymph nodes with epigenetic alterations in CDH1 and RAR-β 2 [24] and hypermethylation of ESR1, BRCA1 and CDH1 in breast LN metastases [25]. Promoter methylation has also been observed in a wide variety of essential molecular pathways in the context of metastatic breast cancer, including genes involved in apoptosis [26], DNA repair [27,28], the regulation and composition of the extracellular matrix [29,30], transcription [31,32] and the cell cycle [33,34]. In addition, epigenetic silencing of the lysosomal cysteine protease inhibitor cystatin 6 (CST6) is more frequently observed in metastatic lesions than in primary cancers [35] and the epigenetic silencing of the chemokine CXCL12 (rather than its receptor CXCR4) contributes to the metastatic potential of mammary carcinoma cells [36]. In contrast with the repressive effects of promoter hypermethylation, hypomethylation events can lead to gene overexpression that can significantly stimulate breast cancer progression and metastasis [37] as well as being an effective molecular indicator of distant metastases [38].

Despite these various reports, no systematic assessments have been performed to identify genome-wide DNA methylation signatures related to models of breast cancer metastasis. Here we describe the first use of promoter tiling microarrays to identify whole-genome epigenetic changes associated with breast cancer metastasis to lymph nodes. We used a highly metastatic variant (MDA-MB-468GFP-LN; 468LN) of the poorly metastatic MDA-MB-468GFP human breast adenocarcinoma cell line [39]. In breast cancer, the lymphatic system serves as a direct route for the spread of primary tumor cells to the lymph nodes and is also a potential route for dissemination throughout the body to distant organs. This variant 468LN cell line displays profound morphological changes and increased growth rate relative to its parental line, and when injected orthotopically into nude mice, these cells produce abundant spontaneous lymph node metastases [39]. We identified widespread hypomethylation and hypermethylation events in these cells by using promoter tiling microarrays and we identified the altered DNA methylation status of several genes implicated in epithelial–mesenchymal transition (EMT) [40]. Furthermore, we confirmed functional associations between this altered methylation status and changes in gene expression with quantitative real-time RT-PCR (qRT-PCR). Here we show for the first time that genome-wide epigenetic alterations may contribute to metastasis through the lymph nodes and that these epigenetic changes are functionally associated with metastatic mechanisms such as EMT.

## Materials and methods

### Cell lines

MDA-MB-468GFP (468GFP) and 468LN cell lines were isolated and characterised as described previously [39] and grown for four passages from frozen stocks in α-minimum essential medium (Invitrogen, Burlington, ON, Canada) supplemented with 10% fetal calf serum (Wisent Inc., St Bruno, Quebec, Canada). At the fifth passage, each cell line was split into three parallel flasks designated biological replicate 1, 2 and 3, and grown to approximately 70% confluence. All experimental research reported in this article was performed within the Safety and Ethical guidelines of the University of Western Ontario.

### Digestion of purified genomic DNA with *Mse*I and ligation to annealed adaptor primers

**Figure 1:**
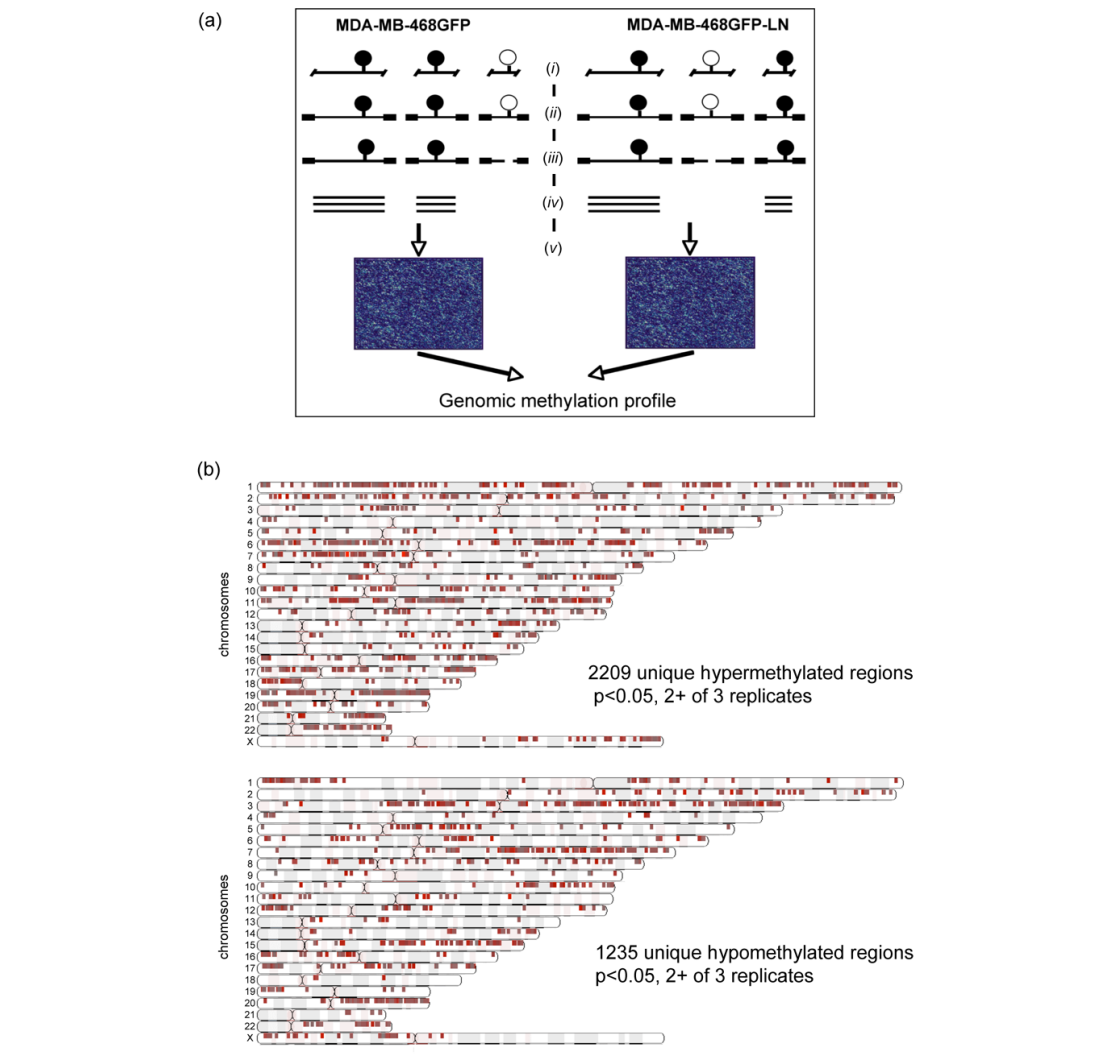
Experimental design and chromosomal mapping of variably methylated targets. **(a) **Experimental design of methylation analysis with Affymetrix human promoter 1.0R microarray. In this adaptor-mediated, PCR-based approach to probe the microarrays, *(i) *DNA was digested with *Mse*I, purified and then *(ii) *adaptors were ligated before *(iii) *a second digestion was performed with the methylation-sensitive *Hha*I enzyme. *(iv) *Samples were then PCR amplified and *(v) *these PCR products were fragmented, labeled, and hybridized to the Affymetrix Human Promoter 1.0R GeneChips. Open circles, unmethylated cytosines; filled circles, methylated cytosines. **(b) **The chromosomal location of variably methylated promoter array gene targets in the MDA-MB-468GFP-LN cells relative to the parental cell line are shown as red blocks and represent significant (*P *< 0.05) targets identified in at least two of three replicates.

An adaptor-mediated, PCR-based approach was used to produce labeled targets for microarrays (Figure 1a). For each cell line, 2 μg of genomic DNA was isolated with the GenElute Genomic DNA Miniprep kit (Sigma-Aldrich, St Louis, MO, USA) in accordance with the manufacturer's instructions and was digested with 10 U of *Mse*I (New England Biolabs, Pickering, ON, Canada) in 1 × NEB2 buffer in a final volume of 50 μl for 3 hours at 37°C. *Mse*I-digested genomic DNA was then purified with the Qiaquick PCR Purification kit (Qiagen, Mississauga, ON, Canada) and eluted in 50 μl. H-12 and H-24 primers were annealed as follows: 4 nmol of H-24 (5'-AGG CAA CTG TGC TAT CCG AGG GAT-3'; Sigma-Genosys, Oakville, ON, Canada) and 4 nmol of H-12 (5'-TAA TCC CTC GGA-3'; Sigma-Genosys) were combined in a final volume of 16 μl, heated to 80°C for 5 minutes and then allowed to cool slowly to 20°C. The annealed primers were then combined with the *Mse*I digested, purified genomic DNA, 5 U of T4 DNA ligase (Invitrogen) and 1 × ligase buffer in a final volume of 80 μl, and the samples were ligated overnight at 16°C. The adaptor-ligated genomic DNA was later purified with the Qiaquick PCR cleanup kit, eluted in 30 μl, digested with *Hha*I in a 50 μl volume for 3 hours at 37°C and then purified. Regions of differential methylation in DNA from both cell lines were determined by comparing signal intensities on microarrays.

### PCR amplification of adaptor-ligated, *Hha*I-digested genomic DNA

To generate amplicons for labeling hybridization to microarrays, the purified adaptor-ligated, *Hha*I-digested DNA was amplified in 50 μl with 1 U of *Taq *polymerase (Invitrogen), 1 × reaction buffer, dATP, dCTP and dGTP (each at 200 μM), 160 μM dTTP, 40 μM dUTP and 1.0 μM H-24 primer. The reaction profile consisted of the following: 72°C for 5 minutes, followed by 25 cycles of 94°C for 1 minute, 55°C for 1.5 minutes and 72°C for 2 minutes, with a final extension step of 72°C for 10 minutes. PCR reactions were then purified as above and eluted in 50 μl. For each biological replicate, 7.5 μg of amplified DNA was fragmented, labeled, and hybridized to Human Promoter 1.0R GeneChips (Affymetrix, Santa Clara, CA, USA), and the arrays were washed and scanned in accordance with the manufacturer's standard protocol. Array Image analysis and spot quantification were performed with Affymetrix GeneChip Operating Software (GCOS) software from Affymetrix. All microarray analyses were performed at the London Regional Genomics Centre and all sequencing was performed at the Robarts Sequencing Facility at the University of Western Ontario.

### Microarray data analysis

The .CEL files (raw methylation measurements generated by GCOS) were then imported into the Partek Genomic Suite Software [41-43]. The imported data were normalized with the Robust Multichip Averaging algorithm [44] and converted to log_2 _values. To detect hypermethylated regions, the mean signal from each probe for the 468GFP cells was subtracted from that of the 468LN cells across all 4.2 million probes. All probes with a positive signal after this subtraction represented regions of increased signal in 468LN cells. (A reverse subtraction was performed to detect regions of significant hypomethylation.) Statistical parameters were set at *P *< 0.05 (single-sided *t *test), with a window of 250 nucleotides, to detect significant regions present in at least two of three biological replicates. These regions were annotated to their corresponding genes with the use of the Probeset ID annotation file from the Affymetrix U133_Plus_2 Expression Array. The 'Chromosome View' tool of the Partek Genomic Suite software visualized hypermethylation/hypomethylation across an entire chromosome; to visualize methylation events at specific promoters, the 'Region View' tool of this software was used to create custom tracks (.wig files) for visualization in the University of California at Santa Cruz (UCSC) Blat genome browser [45].

The data discussed in this publication have been deposited in the National Center for Biotechnology Information's Gene Expression Omnibus (GEO) and are accessible through GEO Series accession number GSE12122.

### Bisulfite genomic sequencing

To confirm DNA methylation status [46], genomic DNA (2 μg) was treated with bisulfite using the Epitect DNA bisulfite treatment kit (Qiagen). Primers specific to the converted DNA were designed with the MethPrimer software [47], using the default parameters for 200 and 500 base-pair amplicons. PCR was performed with 60 ng of DNA in 1 × buffer, 200 μM dNTPs, 2.0 to 2.5 mM MgCl_2_, 400 nM forward and reverse primers (Sigma-Genosys), and 1 U of *Taq *polymerase. The cycling conditions used were asa follows: 1 cycle of 94°C for 5 minutes, followed by 5 cycles of 94°C for 1 minute, 55°C for 2 minutes and 72°C for 2.5 minutes, followed by 35 cycles of 94°C for 1 minute, 55°C for 1 minute and 72°C for 1.5 minutes. PCR products were purified from agarose gels with the Qiaquick PCR Purification kit, and 25 ng of purified product was ligated overnight at 14°C into the T-vector PCR2.1 (Invitrogen) in accordance with the manufacturer's instructions. Plasmids were transformed into TOP10 competent bacteria, and transformed bacteria were spread onto Luria–Bertani agar plates containing 100 μg/ml ampicillin and 50 μl of 10 mg/ml X-gal (5-bromo-4-chloro-3-indolyl-β-D-galactopyranoside) and incubated overnight at 37°C. Potential clones were directly screened by PCR with the gene-specific primers, and clones showing the expected band size were inoculated into 2 ml of LB containing 100 μg/ml ampicillin and grown overnight at 37°C. Plasmid DNA was isolated with the Genelute Plasmid Miniprep kit (Sigma, Oakville, ON, Canada) and sequenced with the T7 promoter primer, and cloned sequences were analyzed by using the ClustalW alignment algorithm [48].

### Quantitative real-time RT-PCR

Total RNA was extracted from three biological replicates of each cell line by using Trizol reagent (Invitrogen), and cDNA was synthesized with Superscript II (Invitrogen) in accordance with the manufacturer's instructions. Real-time primers were designed with Primerquest Software [49]. Reactions in triplicate for each biological replicate used 1 × Brilliant SYBR Green QPCR Master mix (Stratagene; VWR, Mississauga, ON, Canada), 150 nM forward and reverse primers, 200 μM dNTPs and cDNA derived from 100 ng of RNA. For each gene, standard curves were generated by using cDNA derived from 100, 33.3, 11.1, 3.7 or 1.2 ng of total RNA. For each biological replicate, relative amounts of each gene were determined by comparison with the standard curve. The 'unknown' samples were then normalized to glyceraldehyde-3-phosphate dehydrogenase, and the expression levels in the control 468GFP cells were normalized to 1 so that results could be presented as a percentage of fold change, relative to the control.

### Ingenuity Pathways Analysis

Gene networks and canonical pathways representing key genes were identified using the curated Ingenuity Pathways Analysis (IPA) database [50]. The data set containing gene identifiers and corresponding fold changes was uploaded into the web-delivered application and each gene identifier was mapped to its corresponding gene object in the Ingenuity Pathways Knowledge Base (IPKB). The functional analysis identified the biological functions and/or diseases that were most significant to the data sets. Fisher's exact test was performed to calculate a *P *value determining the probability that each biological function and/or disease assigned to the data set was due to chance alone. The data set was mined for significant pathways with the IPA library of canonical pathways, using either (1) a ratio of the number of genes from the data set that mapped to the pathway divided by the total number of genes that mapped to the canonical pathway or (2) a Fisher's exact test to calculate a *P *value determining the probability that the association between the genes in the data set and the canonical pathway was explained by chance alone. In addition, networks were generated by using IPA as graphical representations of the molecular relationships between genes and gene products. The intensity of genes (node) colour in the networks indicates the degree of hypermethylation (blue) or hypomethylation (yellow). Nodes are displayed using various shapes that represent the functional class of gene products.

## Results

### Variably methylated regions are spread across the genomes of 468GFP and 468LN cells

We undertook high-resolution DNA methylation profiling with the Affymetrix human promoter microarray platform to identify metastasis-related methylation differences between the 468GFP and 468LN cell lines. This single-chip technology is composed of more than 4.6 million 25-base-pair probes tiled across 10 to 12.5-kilobase regions and includes the transcription start sites of more than 25,500 gene promoter sequences. DNAs from the cell lines were used in an adaptor-mediated, PCR-based approach to probe triplicate microarrays. In the 468LN cells, relative to the 468GFP cells, we identified 2,209 unique hypermethylated and 1,235 unique hypomethylated regions in at least two of three replicates (*P *< 0.05; Figure 1b). These regions were spread across the genome, and clustering of the methylation changes could be identified across specific chromosome arms. For example, enriched regions of hypermethylation events were identified on chromosomes 6p, 7p, 11p/q, 18p and 19p/q, and similar hypomethylated regions also could be identified (1p, 3q, 7q and 20q). These regions were not uniform, however, because there was evidence of interspersed hypermethylation and hypomethylation events across many of the chromosomes.

**Figure 2:**
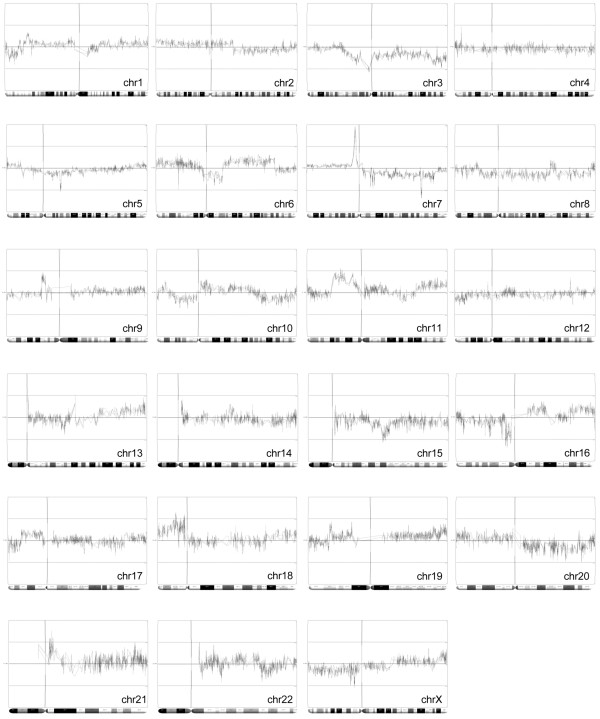
Mapping of genomic DNA methylation changes. Epigenetic 'heat' maps were generated of variably methylated targets located on each chromosome (chr*n*). Genes identified above the center lines are hypermethylated in the MDA-MB-468GFP-LN cells relative to the MDA-MB-468GFP cells; those below the center line are hypomethylated. The overlapping traces represent the individual replicates from each microarray.

The Affymetrix promoter microarrays provide a robust platform that can identify gene-specific and regional differences in DNA methylation patterns in this breast cancer metastasis model. We found a high degree of reproducibility across the replicate microarrays, with individual tracings virtually overlapping at the chromosome level (Figure 2). Furthermore, interfacing the Partek analysis software with the Blat tool of the UCSC genome browser showed that signal reproducibility within individual promoter regions was uniform down to the level of individual probe tiles within these promoter sequences (data not shown).

### Ingenuity Pathways Analysis of differentially methylated gene targets

**Figure 3:**
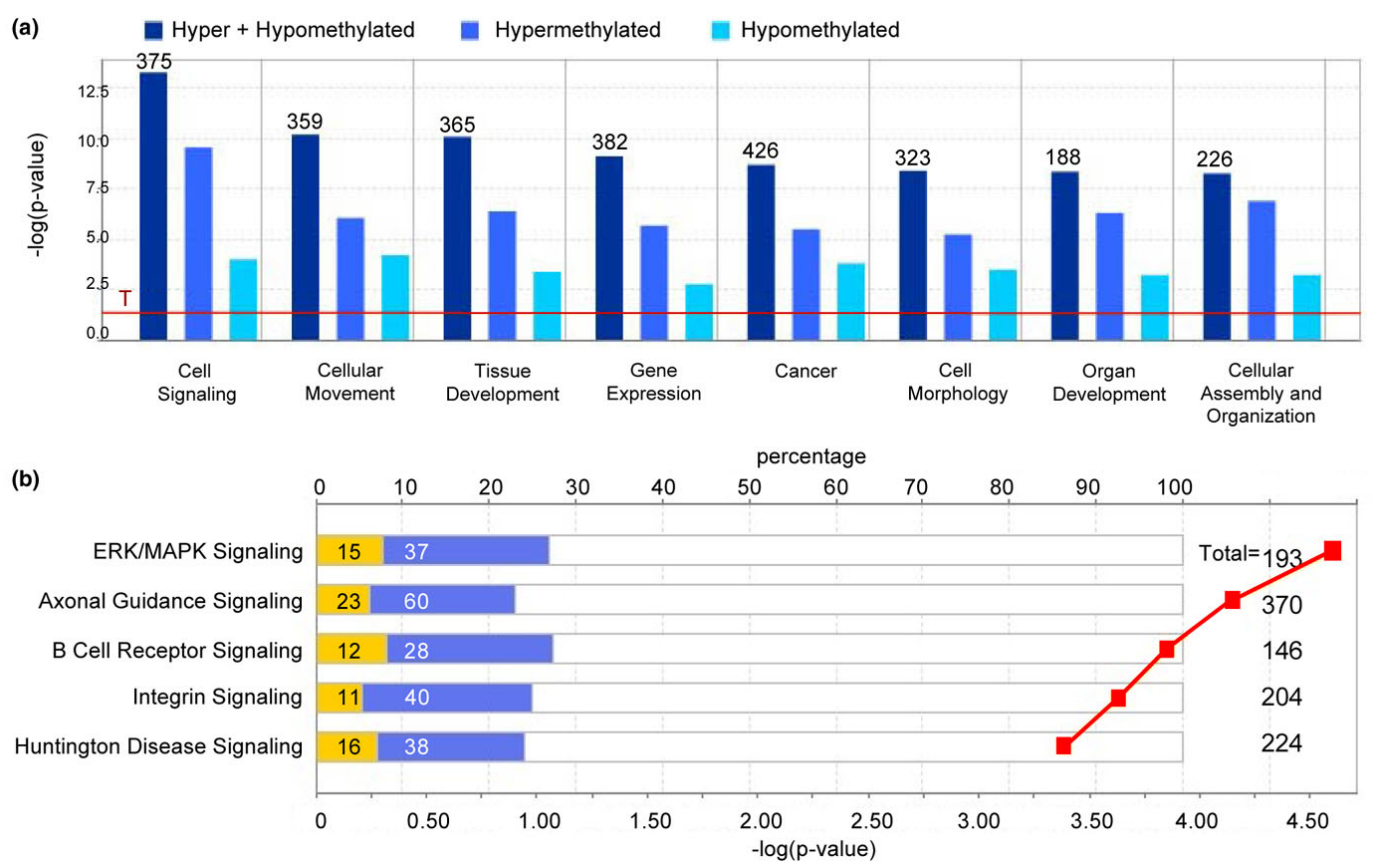
Top functional categories and canonical pathways identified by IPA. **(a) **Top eight functional categories from our data set based on significance. The red line (T) indicates the threshold of – log*P *greater than 2.0. The total numbers of genes (for example 375) and the relative numbers of significant hypomethylated and hypermethylated genes are shown for each category. **(b) **Top five pathways from the Ingenuity Pathways Analysis library of canonical pathways that were most significant to our data set. For each canonical pathway, hypomethylated (yellow) and hypermethylated (blue) genes are shown (for example 15 and 37), as is the total number of genes in that pathway (for example 193). The top axis represents the percentage of genes per pathway, and the bottom axis the significance (red data points).

**Figure 4:**
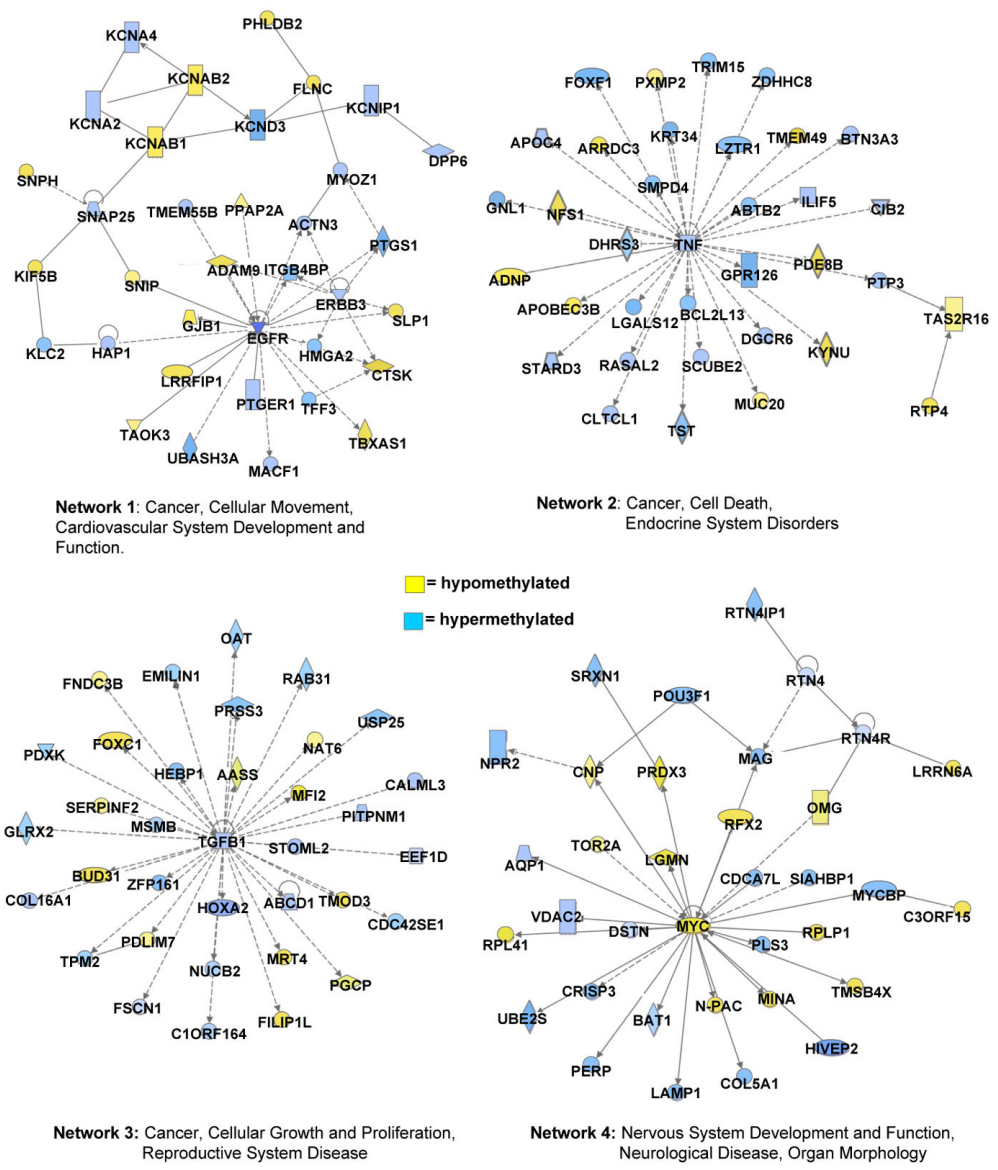
Network diagrams generated as a graphical representation of the molecular relationships between genes and gene products. The gene products are represented as nodes (shapes) and the biological relationship between two nodes is represented as an edge (line). All edges are supported by at least one reference stored in the Ingenuity Pathways Knowledge Base. The intensity of the node color indicates the degree of hypermethylation (blue) or hypomethylation (yellow) and the nodes are displayed using various shapes that represent the functional class of the gene product (the key is given in Figure 5).

Annotated gene lists were created of the 2,209 significantly hypermethylated (*P *< 0.05; Table 1) and 1,235 hypomethylated (*P *< 0.05; Table 2) gene targets identified in the 468LN cells. Although no functional relationships were immediately evident in these lists, the clustering of hypermethylation events at 7p11.1 and our own karyotyping of these cell lines [51] suggest that gene dosage events involving 7p coincide with gene-specific hypermethylation events in this particular region. This may specifically involve chromosomal deletions or duplications that lead to differences in copy number between the two cell lines. We used IPA to investigate the biological relevance of the observed genome-wide methylation changes by categorizing our data set into biological functions and/or diseases (Figure 3a). We determined that promoter hypermethylation was more common than hypomethylation at the genes identified in each of these top eight functional categories. These broad categories each involved between 226 and 375 genes having roles in cell signalling, cellular movement, cancer and other functional categories. We also searched the gene lists to identify significant canonical pathways from the IPA library (Figure 3b). The top five pathways involve signaling pathways in which approximately 30% of the genes display methylation changes. For example, the ERK/MAPK signaling pathway consists of 193 genes, of which 15 were hypomethylated and 37 were hypermethylated. The complete list of all the variably methylated genes identified in these top five pathways is presented in Table 3. Network analysis was also performed to provide a graphical representation of the biological relationships between genes and gene products. The top four networks related to the EGFR, TGFβ 1, TNF and MYC genes, with each network involving approximately 35 hypermethylation and hypomethylation events (Figure 4).

**Table 1:** Top 20 putative hypermethylated gene targets detected by promoter analyses of 468GFP and 468LN cells

Gene name	Common name	Chromosome	Fold change
FK506 binding protein 9, 63 kDa	FKBP9	7p11.1	6.02
EGFR-coamplified and overexpressed protein	ECOP	7p11.2	5.89
Hypothetical protein MGC33530	MGC33530	7p11.2	5.07
LanC lantibiotic synthetase component C-like 2	LANCL2	7p11.2	4.72
Coiled-coil-helix–coiled-coil–helix domain containing 2	CHCHD2	7p11.2	4.55
Epidermal growth factor receptor	EGFR	7p11.2	4.54
FK506 binding protein 9, 63 kDa	FKBP9	7p11.2	3.99
Immunoglobulin heavy locus	IGH@	14q32.33	3.89
ATPase, H^+^, lysosomal 42 kDa, V1 subunit C1	ATP6V1C1	8q22.3	3.86
Beta 1,3-galactosaminyltransferase, polypeptide 1	B3GALNT1	3q25	3.45
Sec61 gamma subunit	SEC61G	7p11.2	3.45
Kelch-like 26 (*Drosophila*)	KLHL26	19p13.11	3.44
phospholipase C, gamma 2	PLCG2	16q24.1	3.41
Ubiquitin specific peptidase 49	USP49	6p21	3.20
Solute carrier family 22, member 6	SLC22A6	11q13.1	3.07
Similar to metallo-beta-lactamase superfamily protein	LOC153364	5q14.3	3.05
Src homology 2 domain containing adaptor protein B	SHB	9p12	3.05
Cholinergic receptor, nicotinic, delta	CHRND	2q33	2.92
Down syndrome critical region gene 9	DSCR9	21q21.13	2.83
Membrane associated ring protein 8	C3HC4	10q11.21	2.75

The names and descriptions of the genes are given, as well as the fold change in hypermethylation in MDA-MB-468GFP-LN (468LN) cells relative to MDA-MB-468GFP (468GFP) cells.

**Table 2:** Top 20 putative hypomethylated gene targets detected by promoter analyses of 468GFP and 468LN cells

Description	Gene symbol	Chromosome	Fold change
Chromogranin A (parathyroid secretory protein 1)	CHGA	14q32	6.44
DKFZP434B0335 protein	DKFZP434B0335	7q21.3	3.73
Zinc finger, C3HC-type containing 1	ZC3HC1	7q32.2	3.41
Centrosomal protein 57 kDa	CEP57	11q21	3.22
Stromal antigen 1	STAG1	3q22.3	3.12
Vesicle-associated membrane protein 3 (cellubrevin)	VAMP3	1q36.23	3.07
Ectonucleoside triphosphate diphosphohydrolase 7	ENTPD7	10q24.2	2.98
Transmembrane emp24 domain trafficking protein 2	TMED2	12q24.31	2.97
Glycoprotein V (platelet)	GP5	3q29	2.96
CATR tumorigenicity conversion 1	CATR1	7q32	2.87
Nuclear receptor subfamily 2, group C, member 2	NR2C2	3p25	2.83
Small EDRK-rich factor 1A (telomeric)	SERF1A	5q12.2-q13.3	2.83
Transcription factor 12	TCF12	15q21	2.82
Zinc finger and BTB domain containing 20	ZBTB20	3q13.2	2.82
Aurora kinase A interacting protein 1	AURKAIP1	1p36.33	2.82
Sorting nexin 1	SNX1	15q22.31	2.82
LOC136263	LOC136263	7q32.2	2.81
NEDD8 ultimate buster-1	NYREN18	7q36	2.77
Testis specific, 13	TSGA13	7q32	2.76
Solute carrier family 3A, member 2	SLC3A2	11q13	2.75

The names and descriptions of the genes are given, as well as the fold change in hypomethylation in MDA-MB-468GFP-LN (468LN) cells relative to MDA-MB-468GFP (468GFP) cells.

**Table 3:** Gene lists identified from the canonical pathway analyses shown in Figure 3b

Canonical pathway	Hypermethylated	Hypomethylated
ERK/MAPK signaling	ARAF, CREB3, CREB5, DUSP4, EGFR, ELF3, FGFR3, FGFR4, FYN, HSPB2, LTK, MERTK, MKNK1, MYCN, PIK3R2, PIK3R3, PIK3R5, PLCG2, PPM1J, PPP1CB, PPP1R10, PPP1R11, PPP1R14A, PPP2R1B, PRKACG, PRKAG2, PRKAR1A, PRKCG, RAC1, RAC2, RAPGEF1, RAPGEF4, ROR2, RPS6KA5, SHC1	ATF4, ELK1, ESR1, FOS, MAPKAPK5, MRAS, MYC, PIK3CA, PLA2G2A, PLA2G6, PPP1R3D, PPP1R7, PRKACA, PRKCD, STAT1
Axonal guidance signaling	ADAM2, ADAM30, ARHGEF12, BAIAP2, BDNF, BMP6, BMP8A, BMP8B, CFL2, DPYSL5, EFNB3, EGFR, EPHA10, EPHA8, EPHB1, FGFR3, FGFR4, FYN, GLI1, GLI2, GNAL, GNAO1, GNAT2, GNG3, L1CAM, LTK, MAG, MERTK, MICAL1, MKNK1, MYL5, MYL7, NFATC1, NFATC4, NGFB, NTF5, PIK3R2, PIK	ABLIM1, ADAM9, BMP15, CDC42, EPHA3, EPHB4, GNAS, KALRN, MRAS, MYL3, NFATC2, NTF3, NTN2L, PIK3CA, PRKACA, PRKCD, ROBO1, SMO, SRGAP3, WASL, WNT16, WNT2
B-cell receptor signaling	BCL10, BCL2A1, BCL3, CALM3, CAMK2B, CARD10, CREB3, CREB5, GSK3A, INPP5D, LYN, MAP2K4, MAP3K11, MAPK13, NFATC1, NFATC4, NFKBIE, PIK3R2, PIK3R3, PIK3R5, PLCG2, POU2F2, PRKCQ, PTEN, RAC1, RAC2, RELA, SHC1	ATF4, BCL6, CDC42, CHUK, ELK1, FCGR2A, FCGR2B, FCGR2C, MAP2K7, MRAS, NFATC2, PIK3CA
Integrin signaling	ACTA1, ACTB, ACTG2, ACTN3, ACTN4, BCAR3, CAPN5, CAPN9, CAPNS1, EGFR, FGFR3, FGFR4, FYN, ITGAX, LTK, MAP2K4, MAP3K11, MERTK, MRCL3, MRLC2, MYL5, MYL7, PIK3R2, PIK3R3, PIK3R5, PLCG2, PPP1CB, PTEN, RAC1, RAC2, RALA, RAPGEF1, RHOC, RHOT2, ROR2, SHC1, TLN1, TN	ARF5, CAV1, CDC42, DDEF1, ITGA10, ITGB2, MRAS, MYLK, PIK3CA, TSPAN3, WASL
Huntington's disease signaling	BDNF, BET1L, CACNA1B, CAPN5, CAPN9, CAPNS1, CREB3, CREB5, DNM3, EGFR, GNG3, HAP1, HSPA1A, HSPA1B, HSPA2, HSPA8, IGF1R, MAP2K4, MAPK13, NAPA, NAPG, NCOR2, NGFB, PACSIN1, PIK3R2, PIK3R3, PIK3R5, POLR2C, POLR2D, POLR2H, POLR2J, PRKCG, PRKCH, PRKCQ, SHC1, SNA	ATF4, CASP2, CASP4, DLG4, DNAJC5, EP300, HDAC7A, MAP2K7, MAPK6, PIK3CA, PRKCD, SDHA, SDHB, STX16, VAMP3, YKT6

### DNA methylation changes in genes involved in epithelial–mesenchymal transition

**Figure 5:**
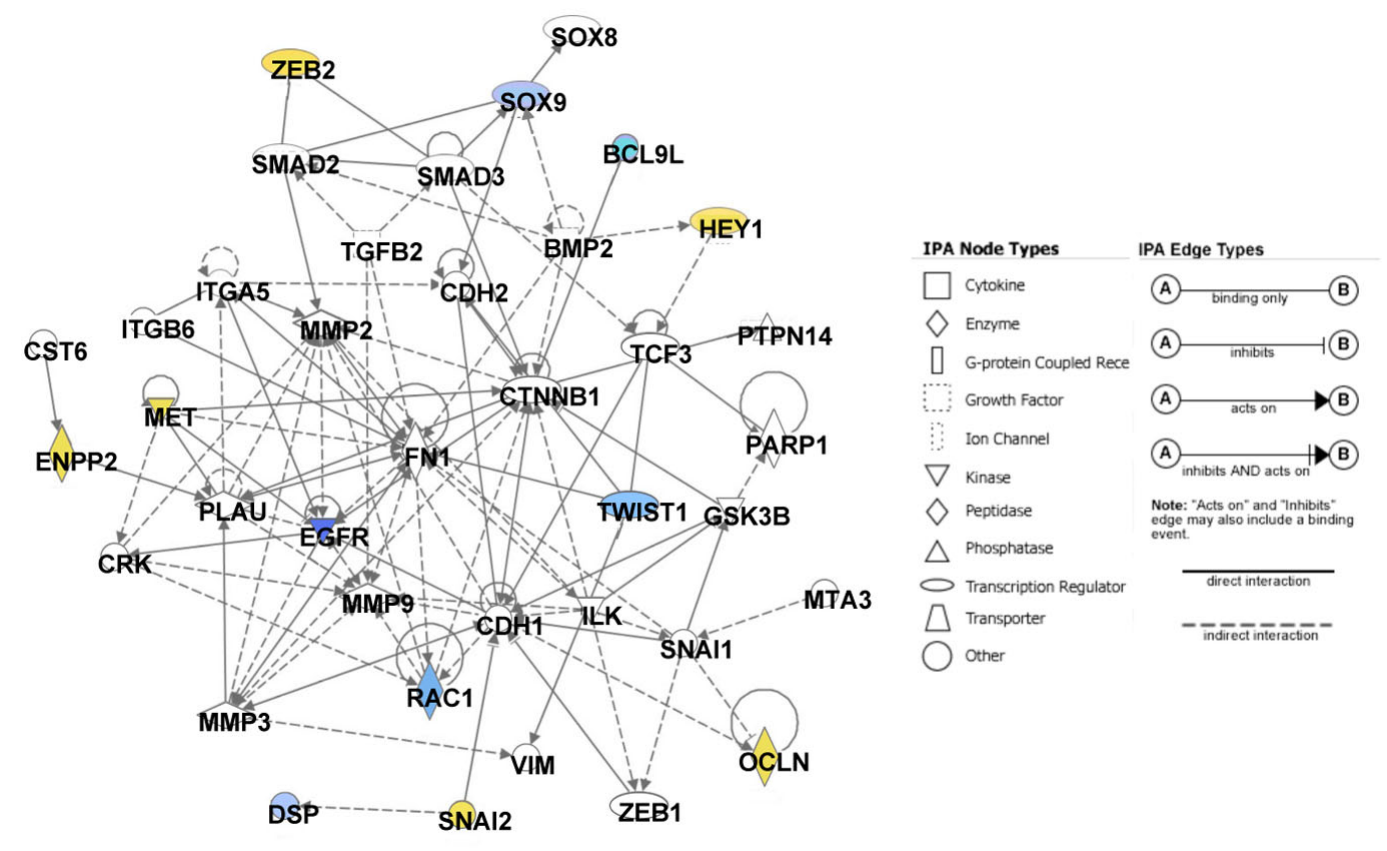
IPA network of genes associated with epithelial–mesenchymal transition. This network diagram shows the biological associations of 35 focus genes associated with epithelial–mesenchymal transition [40] as a graphical representation of the molecular relationships between genes/gene products. The intensity of the node color indicates the degree of hypermethylation (blue) or hypomethylation (yellow) above the significance cutoff and the nodes are displayed using various shapes that represent the functional classes of the gene products as shown in the key.

**Figure 6:**
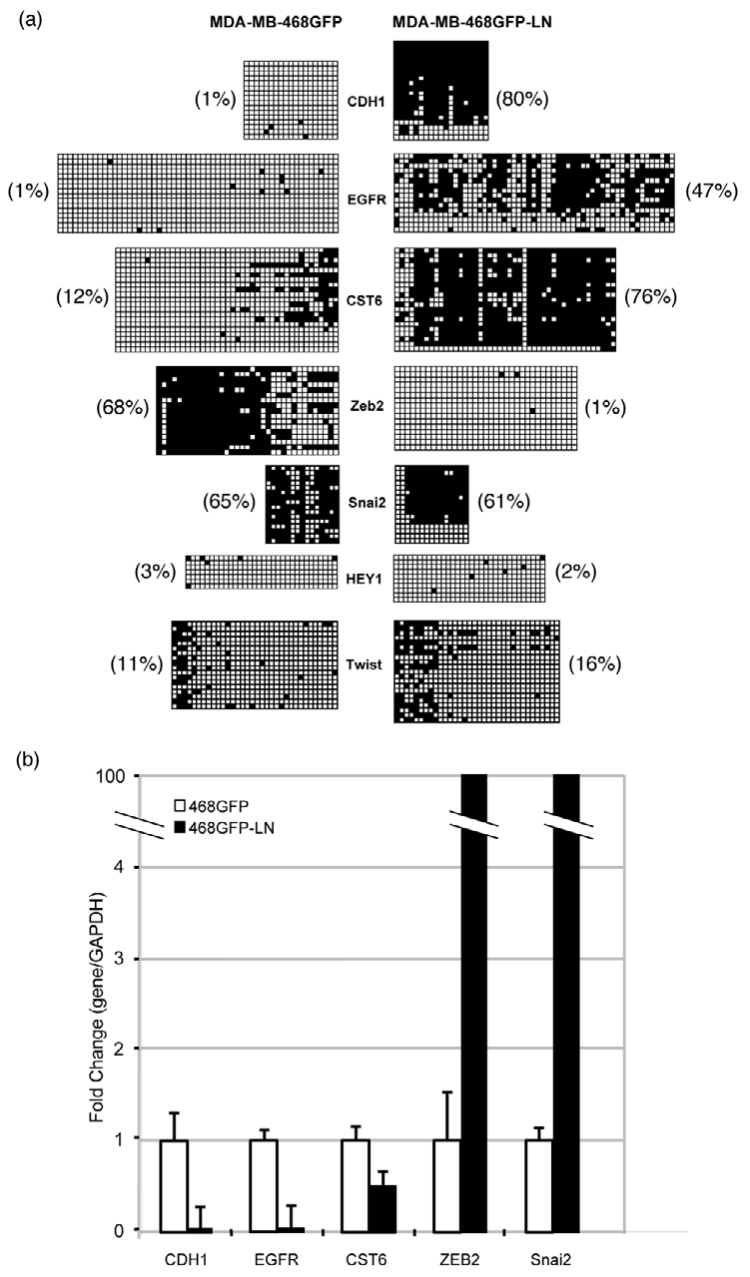
Sodium bisulfite and functional analyses. **(a) **Sodium bisulfite sequencing of representative genes detected with aberrant methylation and/or associated with epithelial–mesenchymal transition. Each square represents a CpG (open square: unmethylated; closed square: methylated). Each row of squares represents one cloned PCR sequence across the gene promoter (about 20 clones were sequenced per gene). Percentages indicate degree of methylation at each gene locus. **(b) **Quantitative real-time RT-PCR expression data for each of these genes.

To complement these database-generated functional analyses, we investigated the involvement of EMT in our breast cancer metastasis model on the basis of our previous observations of distinct morphological differences between these cell lines that showed a shift from an epithelial to a more mesenchymal phenotype [39]. We generated an IPA network that addressed the biological relationships between genes/gene products implicated in EMT [40] and we overlaid our methylation data set onto this network (Figure 5). These genes include commonly used molecular markers of EMT showing both increased and decreased levels of expression that phenotypically increase the capacity for migration, invasion and/or the resistance to apoptosis [40]. Using the statistical parameters described in the Materials and methods section to detect significant changes in methylation status, we undertook sodium bisulfite analysis (Figure 6a) for several genes in this network.

We tested for and identified changes in methylation status at several gene loci, including CDH1, CST6, EGFR and the transcriptional regulators SNAI2 and ZEB2. The EGFR locus was intriguing for several reasons, because we had initially visualized a reproducibly significant hypermethylation 'spike' at the 7p11.2 region, which contains the EGFR gene (Figure 2) as well as several other nearby gene targets on the list of hypermethylated gene targets. Sodium bisulfite analysis using primers specific to the EGFR promoter region (Figure 6a) confirmed the presence of significant levels of DNA methylation within the EGFR promoter in 468LN cells (47% methylated), in contrast with the 468GFP cells (1% methylated). Two other genes (CDH1 and CST6) displayed significant alterations in CpG methylation in the 468LN cells (up to 80% CpG methylation), in contrast with 1 to 12% in the 468GFP cells, confirming the promoter microarray *in silico *analyses. In contrast, ZEB2 was markedly hypomethylated in the 468LN cells, in contrast with the 468GFP cells (1% versus 68% methylation), whereas SNAI2 showed only moderate overall DNA methylation differences (65% versus 61%) between the cell lines. However, these particular bisulfite profiles suggest that two separate SNAI2 allelic methylation patterns (namely hypermethylated and hypomethylated) are present in the 468LN cell line. A similar methylation pattern also seems to be present in the CDH1 clones, which may explain the non-identification of CDH1 by the initial microarray analyses as a significantly hypermethylated target in the 468LN cells.

Quantitative real-time RT-PCR (Figure 6b) showed that expression of the hypermethylated CDH1, CST6 and EGFR genes was decreased in 468LN cells, whereas the hypomethylated ZEB2 gene was significantly increased relative to the 468GFP cells. SNAI2 expression was also significantly expressed in 468LN cells, perhaps reflecting a subpopulation of 468LN cells possessing a hypomethylated SNAI2 allele. Our data suggest that epigenetic mechanisms contribute to the gene expression changes observed with these two cell lines in this breast cancer metastasis model and that these are complex relationships that need to be validated on a gene-by-gene basis.

## Discussion

Previous studies addressing epigenetic contributions to metastasis have primarily focused on mapping increased DNA methylation levels within the promoter regions of individual candidate genes [16,20,21]. These studies have therefore provided only a limited understanding of the functional mechanisms related to metastasis that may be modulated epigenetically, because in most cases only individual candidate genes rather than pathways and/or gene networks have been implicated. Here we have used promoter tiling microarrays and a unique breast cancer metastasis model to provide a whole-genome map of epigenetic changes related to cancer metastasis. The Affymetrix promoter microarray platform provided near-total coverage of CpG-rich regulatory regions in the human genome, with the inclusion of more than 25,000 human promoter regions, including 1,300 cancer-related genes. Our cell-line model system consisted of a poorly metastatic MDA-MB-468GFP human breast adenocarcinoma cell line and a highly metastatic variant (468LN) that exhibits profound morphological changes and an increased growth rate and produces extensive spontaneous lymph node metastases [39].

We directly mapped significant regions of variable DNA methylation to specific CpG islands by interfacing the imported microarray data analyzed by the Partek Genomics Suite software [42] with the UCSC genome browser. We used IPA to mine the large data sets that were generated with Affymetrix promoter microarray platforms and to identify genes belonging to functional categories (Figure 3a) and specific canonical pathways (Figure 3b and Table 3). Functional pathway analysis revealed that multiple epigenetic changes were identified within several broad, all-encompassing biological pathways. Similarly, canonical pathway analysis revealed the involvement of epigenetic targets in signalling pathways linked to metastasis [10,52]. Interestingly, it seems that epigenetic events consistently involve 25 to 30% of the genes in each of these signalling pathways (Figure 3b), suggesting that these epigenetic changes represent a common yet poorly studied mechanism contributing to the metastatic process. In addition, these epigenetically sensitive pathways may represent common therapeutic targets for epigenetic-based chemotherapies that can restore the normal epigenetic and expression patterns of genes [53].

We also identified gene networks displaying epigenetic changes in our breast cancer metastasis model system that may correlate with specific epigenetic/gene-expression changes in the context of lymphatic metastasis. First, we used the curated Ingenuity literature database to identify networks of genes having known biological relationships to each other. As shown in Figures 4 and 5, the top significant networks include both hypermethylated and hypomethylated genes. One network focused on the EGFR gene (Figure 4) and involved 14 hypomethylated and 20 hypermethylated genes, including members of the KCNA family of potassium voltage-gated channel genes and the receptor tyrosine kinase ERBB3. The absence of EGFR methylation in the 468GFP cells confirmed a previous report [54] showing that several breast cancer cell lines, including the parental MDA-MB 468 cells, lack DNA methylation at the EGFR promoter. The hypermethylated status of EGFR in the 468LN cells predicted by the microarray and Ingenuity analysis was verified by sodium bisulfite analysis and furthermore was associated with decreased expression of this gene in comparison with the parental 468GFP cell line (Figure 6). This hypermethylation, along with the repressed EGFR expression that we observe in the 468LN cells, is somewhat paradoxical in that EGFR expression (a known oncogenic characteristic of cancer cells) has apparently been selected against in the more metastatic 468LN cell line.

The three other most significant networks we identified were focused on the TNF, TGFβ 1 and MYC genes. Each of these networks possessed about 36 genes having altered DNA methylation profiles (Figure 4). Our data support previous reports that have also suggested the involvement of epigenetic regulation and/or roles in chromatin remodelling for these genes. For example, the TNF network includes the activity-dependent neuropeptide protein (ADNP), a novel element of SWI/SNF chromatin remodelling complexes that downregulates TNF and may be important in immune surveillance and cancer [55,56]. In addition, concordant epigenetic silencing of TGFβ signaling pathway genes has been widely reported in breast carcinogenesis [57]. MYC overexpression is commonly implicated in breast cancer and metastasis [58], and certain T-cell lymphomas overexpressing MYC possess specific hypermethylation signatures [59].

We also directly addressed molecular mechanisms responsible for the phenotypic characteristics observed in our metastatic breast cancer model. Given the marked morphological differences *in vitro *between these cell lines [39], we mapped the biological association of 35 focus genes reported to be associated with EMT [40]. The resultant network (Figure 5) showed that epigenetic changes could be identified at several of these gene loci, including EGFR, the lysosomal cysteine protease inhibitor cystatin M (CST6) [60] and the transcriptional repressors ZEB2 and SNAI2. SNAI2, Zeb2 and other family members have multiple gene targets and can recruit specific chromatin-remodelling complexes that repress E-cadherin (CDH1), which is frequently downregulated in tumor progression and EMT [61] and implicated in lymph node metastasis [23]. CDH1 did not exceed the initial significance levels set to triage differentially methylated candidate genes, but this gene was nevertheless chosen for analysis given its role as a common target for epigenetic inactivation in metastasis [21] and its functional relationship to other genes in the EMT network. Sodium bisulfite analysis confirmed the methylation status of each of these genes (Figure 6) and we identified high levels of methylation in the CpG-rich promoter regions of CDH1, EGFR and CST6, as well as hypomethylation at the Zeb2 regulatory region, in the 468LN cells. While the promoter microarrays identified significant hypomethylation in the SNAI2 promoter in 468LN cells, bisulfite analysis suggested only modest changes in methylation levels at this gene locus. Interestingly, sodium bisulfite profiles revealed the existence of two populations of SNAI2 clones, one of which was completely demethylated. This suggests that the 468LN cells may include a population of cells in which this gene is hypomethylated and overexpressed. We also determined the functional significance of epigenetic changes observed at these loci by performing qRT-PCR (Figure 6b). The hypermethylated status of CDH1, EGFR and CST6 was associated with significant repression of these genes in the 468LN cells, whereas hypomethylation of Zeb2 was consistent with its upregulation in these cells. We also observed marked upregulation of the other transcriptional repressor SNAI2, despite only limited or modest changes in DNA methylation observed by bisulfite analysis.

Overall, we showed that over 3,400 genes exhibit altered methylation patterns between these two cell lines, with most of the methylation changes observed in the 468LN cells being hypermethylation events (64%). This relatively high frequency of hypermethylation is probably related to technical characteristics of the Affymetrix platform, which used sequences selected from the National Center for Biotechnology Information human genome assembly (Build 34) with repetitive elements removed by Repeatmaster. We also observed that a proportion of these methylation changes seemed to be clustered within particular genomic regions (Figure 1b and Table 1). This suggested to us that either certain chromosomal subregions are hypermethylated (or hypomethylated) in a coordinated manner, or regional gene-dosage events are masking or biasing some epigenetic events at certain loci. Support for this latter possibility comes from G-banding, spectral karyotyping and fluorescence *in situ *hybridization of both cell lines by our group, which has revealed multiple different chromosome aberrations [51]. In particular, our karyotyping analysis showed differences in the modal chromosome number between the cell lines (60 for 468GFP, and 55 for 468LN), as well as the presence of chromosome alterations that are unique to each of the cell lines. For example, the 468GFP cells possessed an isochromosome [i(7)(p10)], whereas this derivative chromosome was absent in the 468LN cell line. In contrast, the 468LN cells possessed a derivative chromosome 8 [der(8);t(8;15)(q22;q24)] that is absent in the parental cell line. Our subsequent methylation analysis in the context of these complex karyotypic differences suggests that at certain loci, methylation detection may be dependent on gene dosage within these specific chromosome regions. For example, our microarray analyses of EMT-related genes identified EGFR and TWIST1 (on the duplicated 7p) as hypermethylated, whereas bisulfite sequencing confirmed the hypermethylated status of only EGFR in the 468LN cells (Figure 6a). In addition, these microarrays predicted SNAI2 and HEY1 (on chromosome 8q11-21) as hypomethylated, with bisulfite sequencing confirming that only SNAI2 was hypomethylated in the 468LN cells.

Our data suggest that the complex genomic reorganization present in cancer cells (for example unbalanced translocations and deletions) may be superimposed over promoter-specific methylation events that may subsequently be responsible for gene-specific expression changes. We believe that it is therefore of critical importance, in such whole-genome epigenetic profiling experiments, to validate promoter methylation status at specific loci by bisulfite sequencing or similar methods. In addition, given the complex relationship between both genetic and epigenetic mechanisms in initiating and maintaining the steps involved in metastasis [11,23], such experiments to validate methylation profiles should be performed concurrently with gene expression studies, to rule out the presence of false positives (or negatives) and to ensure that methylation effects directly repress gene promoter/enhancer regions. Finally, we propose that the concurrent use of either comparative genomic hybridization or single nucleotide polymorphism/copy-number variation microarrays may be warranted to correlate epigenetic and gene expression patterns with differences in gene copy number between cancer cell lines. Such an approach would permit multi-platform analyses that link together genetic, epigenetic and genomic contributions to cancer progression.

## Conclusion

Our use of promoter microarray technology provides a powerful whole-genome approach with which to identify specific epigenetic events that may correlate with particular steps in metastatic progression, such as EMT. This approach will also allow the development of epigenetic signatures of metastasis to be used concurrently with genomic signatures to enable better mapping of the evolving molecular landscape on which metastasis occurs. As a result, diagnostic, prognostic and therapeutic markers that correlate epigenetic and genetic changes can be identified. One important caveat relates to the importance of validating specific epigenetic changes by using alternative methods of methylation analysis as well as the necessity to link specific epigenetic changes functionally with gene expression. In this manner, specific changes in gene expression such as those that we identify as being associated with EMT will permit translational approaches to target altered molecular pathways related to metastatic progression in these cells.

## Abbreviations

468GFP = MDA-MB-468GFP breast adenocarcinoma cells; 468LN = MDA-MB-468LN breast adenocarcinoma cells; EMT = epithelial–mesenchymal transition; IPA = Ingenuity Pathways Analysis; PCR = polymerase chain reaction; qRT-PCR = quantitative real-time RT-PCR; RT = reverse transcriptase; UCSC = University of California at Santa Cruz.

## Competing interests

The authors declare that they have no competing interests.

## Authors' contributions

DR designed and coordinated the studies and wrote and revised the manuscript. JA performed the microarray, bisufite and expression studies and along with BS performed the data analysis. AM performed bisulfite analyses and WK designed and performed the Ingenuity Pathways Analyses. ABT and AFC contributed to study design. All authors read, assisted in revision and approved the final manuscript.
